# Prognostic factors for recovery following acute lateral ankle ligament sprain: a systematic review

**DOI:** 10.1186/s12891-017-1777-9

**Published:** 2017-10-23

**Authors:** Jacqueline Yewande Thompson, Christopher Byrne, Mark A. Williams, David J. Keene, Micheal Maia Schlussel, Sarah E. Lamb

**Affiliations:** 1 Nuffield Department of Orthopaedics Rheumatology and Musculoskeletal Sciences, University of Oxford, Oxford, UK; 2 School of Health Professions, Faculty of Health and Human Sciences, Plymouth University, Plymouth, UK; 3 Department of Sport, Health Sciences and Social Work, Oxford Brookes University, Oxford, UK

**Keywords:** Acute lateral ankle sprain, Prognostic factors, Recovery, Epidemiology, Systematic review

## Abstract

**Background:**

One-third of individuals who sustain an acute lateral ankle ligament sprain suffer significant disability due to pain, functional instability, mechanical instability or recurrent sprain after recovery plateaus at 1 to 5 years post injury. The identification of early prognostic factors associated with poor recovery may provide an opportunity for early-targeted intervention and improve outcome.

**Methods:**

We performed a comprehensive search of AMED, EMBASE, Psych Info, CINAHL, SportDiscus, PubMed, CENTRAL, PEDro, OpenGrey, abstracts and conference proceedings from inception to September 2016. Prospective studies investigating the association between baseline prognostic factors and recovery over time were included. Two independent assessors performed the study selection, data extraction and quality assessment of the studies. A narrative synthesis is presented due to inability to meta-analyse results due to clinical and statistical heterogeneity.

**Results:**

The search strategy yielded 3396 titles/abstracts after duplicates were removed. Thirty-six full text articles were then assessed, nine of which met the study inclusion criteria. Six were prospective cohorts, and three were secondary analyses of randomised controlled trials. Results are presented for nine studies that presented baseline prognostic factors for recovery after an acute ankle sprain. Age, female gender, swelling, restricted range of motion, limited weight bearing ability, pain (at the medial joint line and on weight-bearing dorsi-flexion at 4 weeks, and pain at rest at 3 months), higher injury severity rating, palpation/stress score, non-inversion mechanism injury, lower self-reported recovery, re-sprain within 3 months, MRI determined number of sprained ligaments, severity and bone bruise were found to be independent predictors of poor recovery. Age was one prognostic factor that demonstrated a consistent association with outcome in three studies, however cautious interpretation is advised.

**Conclusions:**

The associations between prognostic factors and poor recovery after an acute lateral ankle sprain are largely inconclusive. At present, there is insufficient evidence to recommend any factor as an independent predictor of outcome. There is a need for well-conducted prospective cohort studies with adequate sample size and long-term follow-up to provide robust evidence on prognostic factors of recovery following an acute lateral ankle sprain.

**Trial registration:**

Prospero registration: CRD42014014471

**Electronic supplementary material:**

The online version of this article (10.1186/s12891-017-1777-9) contains supplementary material, which is available to authorized users.

## Background

Ankle sprains account for the majority of ankle injuries and therefore represent one of the most common musculoskeletal injuries. The incidence rate in the United States general population is 2.15 per 1000 person-years, with sporting activity accounting for half of all injuries [1]. In the Netherlands, an incidence rate of 37.5 and 17.5 per 1000 person-years during sporting activities and activities of daily living respectively was reported over a 10-25 year period [2]. It is estimated that ankle sprains account for up to 1.5 million visits to UK emergency departments each year [3]. A recent systematic review and meta-analysis of the ankle sprain literature, estimated an incidence rate of 11.6 per 1000 exposures and a prevalence of 11.9% [4].

A key feature of acute lateral ankle ligament sprain (ankle sprain) is that about one-third of injured individuals will experience long-term residual symptoms [5–7]. For example, in an observational study of 648 individuals with an ankle sprain, 32% reported chronic complaints of pain, swelling, or recurrent sprains at 7 years [8]. Similarly, 30% of individuals at 2.5 to 5 years post ankle sprain reported pain on activity [9] with one study reporting that 74% of individuals exhibited at least one residual symptom of either pain, swelling, weakness, or instability 1 to 4 years after an ankle sprain [10]. Furthermore, there is evidence to suggest that these long-term residual impairments of the ankle influence an individual’s level of functioning during sporting activities and activities of daily living [6, 8].

The combination of a high volume injury with poor prognosis in one-third of injuries, suggests that being able to predict those individuals with expected poor recovery would be of considerable value to injured individuals and healthcare providers. However, prognostic factors associated with chronic residual symptoms from acute lateral ankle ligament sprains are poorly understood [7]. Understanding prognostic factors for poor recovery following an ankle sprain could help clinicians identify patients with poor prognosis and direct the provision of targeted treatment. Conversely, identifying those patients with good prognosis could have benefits for health care cost and resource use as the most effective treatment for this population is unknown.

Conventional management of ankle sprains which initially begin with instructions to protect and rest the joint, and reduce swelling, and progress to early mobilisation with external support and exercises, has been shown to be beneficial [11–14]. However, studies investigating the addition of a supervised programme of physiotherapy to conventional care found no important clinical difference in outcomes of recovery [13, 14]. Research into prognostic factors of recovery could enable patients on a good recovery trajectory to be distinguished from those who are likely to experience difficulties and better target monitoring and interventions after injury. Therefore, the aim of this review was to systematically review and identify evidence of prognostic factors associated with poor recovery following acute lateral ankle ligament sprain.

## Methods

This systematic review is reported according to PRISMA guidelines [15] and details of the protocol were registered on PROSPERO and can be accessed at https.//www.crd.york.ac.uk/PROSPERO/display_record.asp?ID=CRD42014014471.

Electronic searches were performed from inception to September 2016 in AMED, EMBASE, and Psych Info via Ovid; CINAHL and SportDiscus (EBSCOHost); PubMed, and the Cochrane Register of Clinical Trials using the National Institutes of Health Medical Subject Headings where appropriate. In addition, search strings of health condition or body region were used in the Physiotherapy Evidence Database, International Foot and Ankle Biomechanics, International Ankle Symposium, and Open Grey. No language restrictions were applied in the searches. The bibliographies of all full-text articles included for data extraction were screened for further eligible articles. Details of the search strategy are available in Additional file 1: Appendix A.

Articles were included in this review if they met the following eligibility criteria. (1) The study sample or a separately analysed sub-group had a clinical diagnosis of acute (≤ 7 days) lateral ankle ligament sprain assembled within 7 days of injury; (2) the study had a prospective or retrospective longitudinal design, with at least one follow-up time point and; (3) the study presented data on the effect of at least one baseline prognostic factor on recovery outcomes which are collected at presentation. Studies that included patients with ankle fracture (excluding flake fracture <2 mm), or other recent (< 3 months) lower limb injuries and presented results using descriptive or correctional statistics alone were excluded.

The title and abstract of all records identified by the search strategy were screened by two reviewers (CB, JT) applying the eligibility criteria. A third reviewer (MW) screened 10% of the total identified records. We used the Rayyan systematic review web application during the screening process [16]. Full-text articles of all records eligible for inclusion were independently reviewed by the two reviewers (CB, JT) applying the eligibility criteria and screening for duplication. Any discrepancies between the two independent reviewers regarding eligibility were resolved by consensus or consultation with a third member of the review team (MS or MW). We also made attempts to contact the original authors via electronic mail when supplementary information was required to improve clarity. For all articles eligible for inclusion, both reviewers (CB, JT) independently completed a full data extraction form and a risk of bias assessment form. Following this, the two reviewers met to cross-validate data extraction forms for discrepancies and to reach consensus on risk of bias assessment.

We employed the Quality In Prognosis Studies (QUIPS) tool to assess the risk of bias in the included articles [17]. The QUIPS tool considers six important domains affecting validity and risk of bias in studies of prognostic factors. 1) Study participation, 2) study attrition, 3) prognostic factor measurement, 4) confounding measurement and 5) outcome measurement, and 6) analysis and reporting [17]. The first domain - Study Participation addresses the representativeness of the study sample, i.e. whether the studies reported associations that are valid estimates of the true relationship between the prognostic factor and the outcome of interest in the source population. Here we considered the information provided on the baseline characteristics of study participants to evaluate the risk of selection bias. The second domain - Study Attrition addresses whether participants with follow-up data represent persons enrolled in the study i.e. whether the reported association between the prognostic factor and outcome was biased by the assessment of outcomes in a selected group of participants who completed the study. We sought after reasons for loss to follow-up, and attempts to restrict attrition to ≤20% and reduce the risk of systematic differences in the associations reported. The third and fourth domains respectively, were Prognostic Factor Measurement and Outcome Measurement. These domains address the adequacy of prognostic factor and outcome measurement, i.e. whether the study measured the prognostic factor or outcome in a similar, valid, and reliable way for all participants. In this domain, we sought for similarities in the methods and settings used to reduce mis-classification bias.

The fifth domain, Study Confounding addresses potential confounding factors, i.e. whether another factor may explain the reported association. At this point, we sought after a clear definition of important potential confounding variables a prior, similarities in their measurements and appropriate adjustment for these factors in the analysis. Finally, the sixth domain, Statistical Analysis and Reporting address the appropriateness of the study’s statistical analysis and completeness of reporting i.e. whether results are likely to be spurious or biased because of poor analytical strategies or reporting standards [17]. As a part of the assessment of the adequacy of the approach used in the analysis, we paid particular attention to strategies used to develop the model. These include investigations to check that key assumptions were met, interaction tests that assess the correlation between factors, and performance measures for model diagnosis. For example, we rated studies down when reports on multi-collinearity were not performed or explicit in the study results. For each study, two independent assessors (CB, JT) judged the risk of bias for each of the six domains as low, moderate, or high based on three-to-seven sub-item reporting prompts which were rated as “yes”, “no”, “partial” or “unsure” [17]. A consensus meeting followed during which the two assessors reached agreement upon judgements for each of the six domains and an overall risk of bias for each study. See details of the QUIPS assessment process here.

We present a narrative synthesis of prognostic factors that have demonstrated a statistically significant relationship with recovery outcomes following acute lateral ankle ligament sprains. We defined the quality of the evidence using set criteria. Prognostic factors were classified as demonstrating strong evidence when consistent findings were identified from at least two high quality articles using different cohorts. For moderate evidence, consistent findings were sought from at least two adequate quality studies using different cohorts. Limited evidence was classified as findings identified in one adequate quality article or at least two low quality articles from different cohorts. Finally, inconclusive evidence was defined as inconsistent findings from one low quality cohort alone or insufficient research.

## Results

**Fig. 1:**
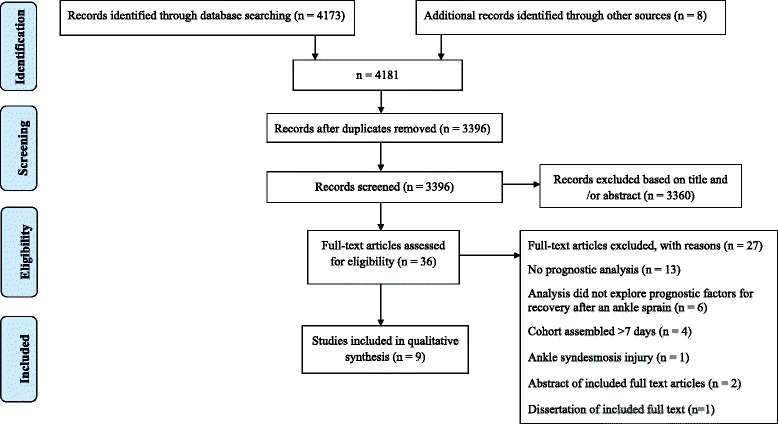
PRISMA flow diagram for the systematic review of prognostic factors for outcome following acute lateral ankle ligament sprain

Figure 1 illustrates the PRISMA flow diagram for this systematic review. The search strategy identified 4173 reports with eight reports identified from additional sources. After removing the duplicate records, the title and abstract of 3396 reports were screened for eligibility. For 3360 reports, the title or abstract clearly indicated that the topic of the report was not relevant to the topic of this review or the reports did not meet our inclusion criteria. The remaining 36 reports were assessed for eligibility as full-text articles. Twenty-seven full-text articles were excluded because they did not employ multivariate prognostic analyses such as linear or logistic regression (*n* = 13) [10, 18–27]; used a cohort assembled >7 days after injury (*n* = 4) [28–31]; used outcome measures that did not meet the study eligibility criteria (*n* = 6) [32–37]; were conference abstracts (*n* = 2) [38, 39] or dissertation (*n* = 1) [40] of full texts already included in the review; or represented participants with ankle syndesmosis injury (n = 1) [41]. Nine studies from nine cohorts were included in the review [42–50].

Table 1 illustrates the key characteristics of the included studies. Six of the nine studies employed a prospective cohort design, whereas three studies [45, 47, 49] represented retrospective analyses of three randomised controlled trials [11–13]. Studies were conducted in five countries. The Netherlands (*n* = 3), USA (n = 3), England (n = 1), Germany (n = 1), and Northern Ireland (n = 1). Five studies employed a single site for recruitment. Settings included school or university sports medicine clinics, hospital emergency departments, primary care Physiotherapists and General Practitioners. A total of 1047 participants, with a median sample size of 33 (range 20-553), provided follow-up data over a time frame ranging from 1 day to 12 months across the studies. Three studies recruited high school or university athletes whereas the remainder recruited from the general population presenting to primary or secondary care. A single large-scale multi-centre randomised controlled trial [12], that recruited participants from eight emergency departments in hospitals across England and demonstrated low risk of bias, accounted for over 50% of the total participants in this review [45]. This study presented only two prognostic factors of recovery – age and female gender.

**Table 1:** Key characteristics of included studies

Study	Design	Setting	Sample size	Sample characteristics	Time since injury	Injury severity	Follow-up
de Bie et al. [42]	Prospective cohort	The Netherlands 1 x Hospital FAD	*N* = 35 at baseline *N* = 33 at 2 weeks *N* = 31 at 4 weeks	General population *N* = 22 M, 13 F 28 ± 10 (13-59) y	NR	NR	2 weeks 4 weeks
Wilson & Gansneder [43]	Prospective cohort	USA 1 x University	*N* = 24 at baseline *N* = 21 at follow-up	Athletes N = 13 M, 8 F 20 ± 2 y	67.8 ± 15.2 h	Grade I, II	11.9 ± 6.6 days
Cross et al. [44]	Prospective cohort	USA 1 x University	N = 20 at baseline *N* = 20 at follow-up	Athletes *N* = 7 M, 13 F 19 ± 1 (18-21) y	≤ 24 h	NR	14.7 ± 8.8 (3–40) days
Akacha et al. [45]	Retrospective cohort	England 8 x Hospital ED	*N* = 584 at baseline *N* = 553 at 4 weeks, 12 weeks, & 9 months	General population *N* = 321 M, 232 F 30 ± 11 (16-72) y	≤ 7 days	Severe (NWB at 3 days)	4 weeks 12 weeks 9 months
Langner et al. [46]	Prospective cohort	Germany 1 x Hospital ED	*N* = 38 at baseline *N* = 26 at 6 months N = NR at 12 months	General population *N* = 18 M, 20 F 38 ± 13 (20-75) y	< 24 h	ATFL Grade I (27%), II (27%), III (46%)	6 months 12 months
van Middelkoop et al. [47]	Retrospective cohort	The Netherlands 32 x GP 1 x Hospital ED	*N* = 102 at baseline *N* = 95 at 3 months *N* = 80 at 12 months	General population *N* = 59 M, 43 F 37 ± 12 (18-60) y	≤ 7 days	Mild (42%), moderate or severe (44%), unknown (14%)	3 months 12 months
van der Wees et al. [48]	Prospective cohort	The Netherlands 20 x Primary care Physiotherapists	*N* = 107 at baseline N = 33 at 2 weeks	General population *N* = 65 M, 42 F 32 ± 14 y	8.7 ± 8.9 days ≤ 5 days *N* = 53 > 5 days *N* = 54	Light (50%), severe (50%)	2 weeks
O’Connor et al. [49]	Retrospective cohort	Northern Ireland 1 x Hospital ED 1 x University sports injury clinic	*N* = 101 at baseline N = NR at 4 weeks *N* = 85 at 4 months	General population, athletes. *N* = 69 M, 31 F 27 ± 10 (16-58) y	< 7 days 40 ± 36 h	Grade I (26%), II (63%), II+ (11%)	4 weeks 4 months
McKeon et al. [50]	Prospective cohort	USA 7 x High schools	*N* = 204 sprains at baseline *N* = 198 sprains in analysis	High school athletes	≤ 24 h	Time to return to play. Same day (23.7%), next day (21.2%), 3 days (29.3%), 7 days (11.6%), 10 days (8.6%), >22 days (5.6%)	Time to return to play. Same day, next day, 3 days, 7 days, 10 days, 21 days, >22 days.

*Abbreviations*: *FAD* first aid department; *N* number; *M* males; *F* females; *y* years; *NR* not reported, *NWB* non-weight bearing status; *ED* emergency department; *ATFL* anterior talofibular ligament; *GP* general practice primary care

**Fig. 2:**
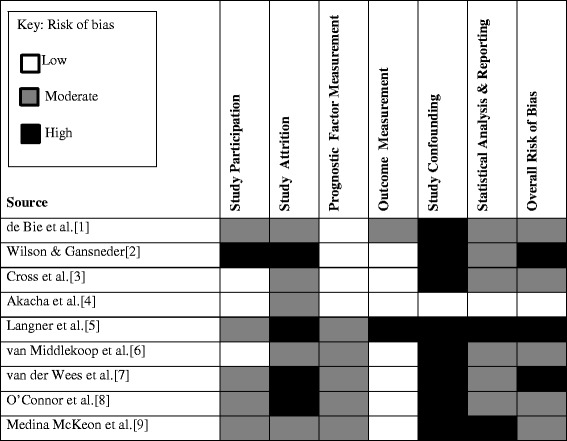
Risk of bias assessment of the nine included studies according to the Quality in Prognosis Studies (QUIPS) tool [10]

Figure 2 illustrates the risk of bias assessment for the nine included studies. See online supplementary information (Additional file 2: Appendix B) for further details. Studies were judged particularly poorly on the risk of bias domains of Study Attrition, Study Confounding, and Statistical Analysis and Reporting. One study was classified as having an overall low risk of bias [45], five having an overall moderate risk of bias [42, 44, 47, 49, 50], and three studies as having an overall high risk of bias [43, 46, 48]. Fig. 2 presents the risk of bias ratings for the prognostic factors identified each time it was explored in a study. The overall quality of evidence derived was mainly from eight studies with high-to-moderate risk of bias (*n* = 8) and one study with low risk of bias (*n* = 1).

Most of the studies rated poorly due to incomplete and/or inadequate reporting standards within individual studies. The main discrepancies we identified were related to the use of poor statistical methods or poor reporting standards. For example, no study reported performing a collinearity diagnostics to check for multi-collinearity between the prognostic factors presented in the final models. In addition, none of the studies included in the review explored or reported results for the performance of their models (measures of interval validity or external validation). The regression analyses employed were not reported in sufficient detail to identify whether prognostic factors were eliminated due to low statistical power or poor clinical utility.

The high loss to follow-up identified in two studies [46, 48] is a pointer to the risk of selection bias that may have been due to the method of recruitment employed. However, these studies did not provide information on the comparisons between participants who completed and who did not complete the final follow-up. Consequently, the profile of the participants lost to follow-up cannot be accurately evaluated. This further highlights the poor reporting standards employed by the studies included in the review.

Meta-analysis was inappropriate due to the heterogeneous nature of prognostic factors, recovery outcome measures, follow-up durations, and the limited number of included studies. Prognostic factors were categorised according to the duration of follow-up employed in the study and grouped as relevant to short term (≤8 weeks), medium-term (≤4 months), and long-term (>4 months) recovery. No factor demonstrated strong evidence of an association with recovery.

### Prognostic factors for short-term recovery (≤8 weeks)

Five of the nine included studies reported data on prognostic factors for short-term recovery [42–44, 48, 49]. Table 2 summarises the analytical approach and prognostic factors identified from these studies. de Bie et al. [42] reported that baseline Ankle Function Score (AFS) ≤ 35 was a prognostic factor for non-recovery at 2 weeks with a sensitivity and specificity of 97% and 100%, respectively. Non-recovery at 4 weeks was predicted by the combination of three baseline prognostic factors (i.e. AFS ≤ 35, higher 0-10 severity grading by a doctor, and higher palpation / ligament stress test score) with a sensitivity and specificity of 81% and 80%, respectively [42]. Similarly, van der Wees et al. [48] using only patients with baseline measurements ≤5 days after injury, reported that baseline AFS ≤ 40 was a prognostic factor for non-recovery at 2 weeks with a sensitivity and specificity of 76% and 63%, respectively. Wilson & Gansneder [43] reported that greater impairment (i.e. greater range of motion loss and greater swelling) were prognostic factors for a longer disability duration (i.e. 11.9 ± 6.6 days) with 34% of the variance in disability duration explained by their combination in a regression model. They also reported that greater functional limitation (i.e. lower scores on an objective six-item weight-bearing activity score and self-reported current athletic ability rating) as prognostic factors for greater disability duration with 33% of the variance explained [43]. The combination of impairment and functional limitation prognostic factors produced an additive effect and explained 59% of the variance in disability duration [43]. Cross et al. [44] reported the baseline prognostic factors of lower self-reported physical function (R2 = .28), lower self-reported global function (R2 = .22), and lower objectively measured ambulation status (R2 = .27) as being associated with a greater number of days to return to sport (i.e. 14.7 ± 8.8 days). When combined into a multiple regression model, the three prognostic factors explained 37% of the variance in number of days to return-to-sport [44].

**Table 2:** Prognostic factors for short-term (≤ 8 weeks) outcome in acute lateral ankle sprain

Study	Primary outcome measure	Independent variables	Analysis	Prognostic factors for short-term outcome
de Bie et al. [42]	Healed or not healed at 2 & 4 weeks. Healed = AFS >75 (0-100) & palpation/ligament stress test score < 2 (0-12).	AFS (0-100) ≤35, doctor severity grading (0-10), palpation/ligament stress test score (0-12).	Multivariate logistic regression	2 weeks: Baseline AFS ≤ 35 predicted recovery status. Sensitivity = 97%, specificity = 100%. 4 weeks: Combined baseline AFS ≤ 35, severity grading, & palpation/ligament stress test score predicted recovery status. Sensitivity = 81%, specificity = 80%.
Wilson & Gansneder [43]	Number of days to return to full sports practice or competition (11.9 ± 6.6 days).	Joint swelling (ml), sagittal plane ROM loss (°), objective WB activity score (0-6), self-reported athletic ability score (VAS, 0-100).	Hierarchical regression	Combined swelling (*β* = −.02) & ROM loss (*β* = −.08). *R* ^2^ = .34, *p* = .023. Combined WB activity score (*β* = −.55) & self-reported ability score (*β* = −.39). *R* ^2^ = .33, *p* = .004. Combined swelling, ROM loss, WB activity score, & self-reported athletic ability score. Adjusted *R* ^2^ = .59; *p* = .001.
Cross et al. [44]	Number of days to return to sport (14.7 ± 8.8 days).	SF36PF (0-100), Self-reported global function (0-100%), objective ambulation status (1-7).	Univariate regression, stepwise multivariate regression.	SF36PF. *R* ^2^ = .28, *p* = .016. Self-reported global function. *R* ^2^ = .22, *p* = .036. Objective ambulation status. *R* ^2^ = .22, *p* = .019. Combined SF36PF, self-reported global function, & objective ambulation status. Adjusted *R* ^2^ = .34, *p* < .01.
van der Wees et al. [48]	Global perceived effect ≥2 (1 = recovered, 2-7 = not recovered) at 2 weeks.	AFS (0-100) ≤40.	Sensitivity & specificity using sample with ≤5 days duration of injury (*n* = 53)	2 weeks: Baseline AFS ≤ 40 predicted recovery status. Sensitivity = 76%, specificity = 63%.
O’Connor et al. [49]	Karlsson function score (0-100) at 4 weeks.	Age (years), injury grade (1, 2, 2+), WB status (FWB, FWB with pain, PWB, NWB).	Univariate regression, step-wise multivariate regression.	4 weeks: Combined age (*β* = −.32, *p* = .001), injury grade (*β* = −.23, *p* = .003), & WB status (*β* = −.34, *p* = .038). Adjusted *R* ^2^ = .34, *p* < .01.

*Abbreviations*: *AFS* ankle function score; ° degrees; *VAS* visual analogue scale; *R* ^2^ the coefficient of determination; *β* standardised beta; *ROM* range of motion; *WB* weight-bearing; *SF36PF* short form-36 physical function scale; *FWB* full weight-bearing status; *PWB* partial weight-bearing status; *NWB* non-weight-bearing status

O’Connor et al. [49] reported that lower subjective ankle function at 4 weeks was significantly associated with the baseline prognostic factors of greater age (*β* = −. 32), more severe injury grade (*β* = −.23), and poorer weight bearing status (*β* = −.34). When combined in a stepwise multivariate regression model, the prognostic factors explained 34% of the variance in subjective ankle function at 4 weeks [49]. Finally, Medina McKeon et al. [50] reported that recurrent ankle sprain was not a prognostic factor in explaining time to return-to-play. They reported no significant difference in Kaplan-Meier time to return-to-play curves for new (median = 3 days, inter-quartile ranges = same day to 7-day return) and recurrent (median = next day, inter-quartile range = next day to 7-day return) ankle sprains [50]. Only two [44, 49] studies explored univariate correlations between variables included in the model. However, overall, measures of functional ability explained larger part of the variance of recovery compared with measures of symptoms of clinical severity alone.

### Prognostic factors for medium-term recovery (≤4 months)

Table 3 shows data reported by one study on prognostic factors for medium term recovery. O’Connor et al. [49] reported that 20% of the variance in subjective ankle function at 4 months was explained by the combined baseline prognostic factors of age, weight bearing status, and injury mechanism. The study participants, who sustained a lateral ankle ligament sprain, were classified as having sustained an injury via an inversion mechanism (70%), or other mechanisms of injury in the analysis [49]. Greater age (*β* = −.26), poorer weight bearing status (*β* = −.23), and non-inversion injury mechanism (*β* = −.25) were prognostic factors for poorer subjective function at 4 months follow-up [49]. The authors also identified medial joint line pain on palpation (*β* = .24) and pain on WB during ankle dorsiflexion (β = .60) at 4 weeks as prognostic factors for poorer subjective function at 4 months [49]. These two independent variables explained 49% of the variance in subjective ankle function at 4 months [49]. Only a small difference was identified in the magnitude of variance explained by measures of severity versus measures of functional ability at presentation of injury. However, at 4 weeks, the ability to weight bear explained a larger percentage of the variance of the model.

**Table 3:** Prognostic factors for medium-term (≤ 4 months) outcome in acute lateral ankle sprain

Study	Primary outcome measure	Independent variable(s)	Analysis	Prognostic factors for medium-term outcome
O’Connor et al. [49]	Karlsson ankle function scores (0-100) at 4 months.	Baseline. Age (years); WB status (FWB, FWB with pain, PWB, NWB); injury mechanism (inversion / other). 4 weeks. Pain on WB ankle DF; medial joint line pain (yes/no).	Univariate regression, step-wise multivariate regression.	4 months: baseline combined age (*β* = −.26, p = .01), WB status (*β* = −.23, *p* = .25), & injury mechanism (*β* = −.25, *p* = .17). Adjusted *R* ^2^ = .34, *p* < .01. 4 months: 4 week combined pain on WB ankle DF (*β* = .60, *p* < .001), medial joint line pain (*β* = .24, *p* = .07). Adjusted *R* ^2^ = .49, p < .01.

*Abbreviations*: *WB* weight-bearing; *FWB* full weight-bearing status; *PWB* partial weight-bearing status; *NWB* non-weight-bearing status; *DF* dorsiflexion, *β*, standardised beta; *R,* ^2^ the coefficient of determination

### Prognostic factors for long-term recovery (>4 months)

Table 4 summarises three studies that reported data on prognostic factors for long-term recovery [45–47]. Akacha et al. [45] employed non-linear mixed modelling to re-analyse data from a large scale RCT [12]. They demonstrated that higher age and female gender were prognostic factors for slower and incomplete recovery [45]. For example, the predicted time to attain a FAOS-S (0-100) score of 65 for 21-year-old male and female participants receiving below knee cast treatment was 2.9 (95% CI. 2.4 to 3.4) and 3.9 (95% CI. 3.0 to 4.7) weeks, respectively [45]. In contrast, 66-year-old male and female participants receiving the same treatment were predicted to attain a score of 65 in 8.3 (95% CI. 4.2 to 12.5) and 17.1 (95% CI. 4.7 to 29.5) weeks, respectively [45]. At 12 months follow-up, Langner et al. [46] reported that three baseline prognostic factors of more severe MRI grading of injury (*R* ^2^ = .45), greater number of injured ligaments determined by MRI (*R* ^2^ = .35), and presence of a bone bruise determined by MRI (*R* ^2^ = .32) were associated with greater time to return to sports activities. Van Middelkoop et al. [47] reported that none of their potential prognostic factors measured at baseline were associated with outcome at 12 months follow-up. Further sub-group analysis of 63 non-recovered participants at 3 months revealed that having a re-sprain within 3 months (*β* = −1.64) and the magnitude of pain at rest at 3 months (*β* = −.69) were prognostic factors for poorer self-reported recovery at 12 months [47].

**Table 4:** Prognostic factors for long-term (> 4 months) outcome in acute lateral ankle sprain

Study	Primary outcome measure	Independent variable(s)	Analysis	Prognostic factors for long-term outcome
Akacha et al. [45]	FAOS-S (0-100, 0 = extreme symptoms, 100 = no symptoms).	Age, gender.	Non-linear mixed model	Greater age and female gender associated with slower and incomplete recovery. Greater age (*β* = −0.01, 95% CI −0.12 to −0.004) Female (*β* = −0.06, 95% CI −0.01 to −0.002)
Langner et al. [46]	Time to return to sports activities.	MRI grading of ligamentous injury (1-3, 1 = stretching, 2 = partial tear, 3 = complete tear); number of injured ligaments; presence of bone bruise.	Multivariate regression	MRI grading of ligamentous injury, *R* ^2^ = .45, *p* < 0.01. Number of injured ligaments, *R* ^2^ = .35, *p* < 0.01. Bone bruise, *R* ^2^ = .32, *p* < 0.01.
Van Middelkoop et al. [47]	Self-reported recovery (NRS, 0-10. 0 = not recovered; 10 = completely recovered) at 12 months.	Re-sprain within 3 months; pain at rest at 3 months (NRS, 0-10).	Multivariate regression	12 months. Re-sprain within 3 months (*β* = −1.64, 95% CI −3.11 to −.16); pain at rest at 3 months (*β* = −.69, 95% CI −1.08 to −.29).

*Abbreviations*: *FAOS-S* foot and ankle outcome score symptoms subscale; *β* standardised beta; *95% CI* 95% confidence interval; *MRI* magnetic resonance imaging; *R* ^2^ the coefficient of determination; *NRS* numerical rating scale

## Discussion

This systematic review provides a summary of prognostic factors of recovery after an acute ankle sprain. Nineteen prognostic factors demonstrated an association with outcome in the final multivariate models presented across the included studies. These measures are mostly acknowledged in the routine management of ankle sprains (See Table 5).

**Table 5:** Summary of number of studies reporting prognostic factors for poor outcome in acute lateral ankle sprain

No	Follow-up time points/Baseline prognostic factors explored	Number of studies reporting an association (n)
1.	At short-term follow-up (≤8 weeks)	
	Age	1 [49]
	Swelling	1 [43]
	Reduced range of motion	1 [43]
	Palpation stress test scores	1 [42]
	Self-reported physical limitations	1 [44]
	Self-reported athletic ability	1 [43]
	Injury severity rating	2 [42, 49]
	Ankle function score	2 [42, 48]
	Weight bearing ability / status	3 [42, 44, 49]
2.	At medium-term (≤4 months)	
	Age	1 [49]
	Non-inversion injury	1 [49]
	Pain (medial joint line) at week 4	1 [49]
	Pain (on WB DF) at week 4	1 [49]
	WB status	1 [49]
3.	At long-term (>4 months)	
	Age	1 [45]
	Female gender	1 [45]
	MRI, severity grading	1 [46]
	MRI, number of ligaments	1 [46]
	MRI, bone bruise	1 [46]
	Pain (at rest) at 3 months	1 [50]
	Re-sprain within 3 months	1 [50]

*Abbreviations*: *WB* weight-bearing; *DF* ankle dorsiflexion; *MRI* magnetic resonance imaging

At short-term follow-up, we found consistent findings from at least two studies with moderate risk of bias, for weight-bearing status and injury grade, indicative of moderate evidence. There was limited evidence for age, pain reproduced by ligament stress test, and the patient reported measures of levels of physical activity. The evidence for swelling, restricted joint range of motion, and self-report athletic ability was inconclusive, due to insufficient findings from two studies with a high risk of bias. This seems to suggest that the severity of the injury and objective assessment of ability to weight-bear demonstrate some degree of accuracy in predicting return to pre-injury functional status.

At medium term follow-up, pain, weight-bearing, mechanism of injury and functional activity score were identified as prognostic indicators of recovery; demonstrating limited evidence from only one study with moderate risk of bias. Similarly, one study [24] included in a review [7] reported high levels of athletic competition, defined as ≥3 times of training per week, as a prognostic factor for poor recovery. However, that study [24] did not adjust for other important prognostic factors or confounding variables such as previous injury.

At long term follow-up, there was limited evidence from one study [45] showing evidence for female gender and age as a prognostic factor for recovery. However, these may be confounded by psycho-social factors such as recovery expectations, coping mechanisms or self-efficacy that have been linked to recovery in musculoskeletal conditions [51]. Other prognostic factors with insufficient evidence for long-term outcome include injury severity, the number of injured ligaments and the presence of bone bruise as determined by magnetic resonance imaging. The observation of insufficient evidence for radiographic findings and recovery, suggests that structural pathology may not be indicative of clinical presentation. The lack of an association between structural changes in the ankle observed with imaging techniques and persistent impairment has also been reported by previous research [52]. It seems that diagnostic classifications may have a poor reliability in predicting recovery at long term. In this review, baseline measures of pain at rest and re-sprain at long-term also showed no association with recovery [47]. This is, however, contrary to reports of an association between recurrent sprains and chronic ankle instability noted by a previous systematic review [53].

Studies with low risk of bias and larger sample sizes tended to report conservative estimates of the association between variables and recovery. For example, the study by Akacha et al. [45] which included over 500 participants reported a *β* value - indicating the amount of change in the rate of improvement expected with one unit change in the prognostic factors when other variables are held constant. The study reported that the maximum achievable score on the foot and ankle outcome score (FAOS) varied over time with greater age explaining 1% and female gender 6% of the variance. In contrast, another study [43] that included only 20 participants reported combined prognostic factors of impairment and function that explained 60% of the variance in recovery. Overall, while the included studies in this review do not provide definite evidence of a causal link between the factors identified and recovery, they do highlight the of role biomechanical factors on recovery.

Overall, a number of the selected prognostic factors identified, demonstrated some consistency across short, medium and long-term recovery time-points. We defined factors as consistent when it was explored by at least two studies or at two different time points within the same study. Measures of pain [42, 47–49], swelling [42, 43, 48], injury severity [42, 46, 49], weight-bearing status [43, 48, 49] and self-reported functional ability [42–44, 48] showed some degree of consistency, however, the evidence of an association with recovery is equivocal because of the poor quality of individual studies. Evidence for the prognostic value of age was, however, consistent according to results from one study with low risk of bias [45], and another study [49] with moderate risk of bias. Higher baseline age was associated with poor recovery at short [49], medium [49] and long term follow-up time points [45].

We observed a trend where clinical indicators of symptoms such as swelling, injury severity, or restricted range of motion (ROM) demonstrated a greater prognostic ability of recovery at short- and medium term, than at long-term follow-up. This may be useful to inform clinical decision making earlier on in the recovery pathway. Measures explored later in the course of recovery, rather than early on, seemed to have a good prognostic value. Examples of these factors include pain at rest, on palpation and on weight bearing, as well as self-reported functional ability. This may suggest that measures of functional ability may be more sensitive at identifying sensory or neuro-muscular deficits in patients experiencing functional or mechanical instability. Alternatively, this may imply that the timing of the measurements, influences association.

To our knowledge, this is the first systematic review of prognostic factors specific to recovery from acute ankle sprains. Overall, results of previous reviews [53, 54] support the findings of our review, and the relevance of these factors to the prediction of recovery in the management of ankle sprains remains conflicting. We observed a substantial amount of clinical and methodological heterogeneity. There were differences in the treatments administered to the study participants, classification of an index ankle sprain (3 studies with inclusion criteria of ≤24 h since injury), injury severity, the duration of follow-up, the measurement instruments employed, and the methodological quality. Furthermore, there was little overlap in the definition of outcome variables and considerable variation across the potential prognostic factors explored in studies. This made the statistical pooling of the results a difficulty. It is worth noting that a significant proportion of the participants included in this review sustained grade I or II injuries, with considerably shorter duration for return to function.

One of the strengths of this review is that we included a homogenous study population of acute lateral ankle ligament injuries, excluding other ligamentous injuries (i.e. peroneal tendon ruptures or high ankle sprain) that predisposes patients to longer recovery trajectory. Furthermore, two-thirds of the sample included in this review were broadly representative of the age range, severity presentations and recreational activity levels of the general population, allowing transferability to most real world acute settings.

There were considerable differences in the measurement of study factors, poorly defined selection procedures for potential prognostic factors, and different outcomes with little or no overlap. For example, injury severity was reported as a prognostic factor associated with recovery, however, two studies used clinical symptoms [42, 49], while a third study [46] used MRI to evaluate grade severity. This made direct comparisons difficult as previous research has shown poor associations between radiographic findings and recovery [53]. Subjective methods increase variability in measurement errors, but objective assessments using MRI are not readily available in acute settings. A number of studies did not use validated outcome measures. For example, two studies [42, 48] used a continuous outcome measure that was dichotomised using an arbitrary cut-off point of ≤35 points to indicate recovery [42] and ≤40 points for a mild ankle injury [48]. There was no pre-specification of this cut-off point from the wider literature; hence, this threshold may not be valid and could have introduced bias.

It has been suggested that a minimum of 10 events may not be required for each prognostic factor considered in a study [55]. However, most of the studies (*n* = 7) included in our review had too small sample sizes in relation to the number of predictors that were explored and tended to be unreliable. Only one study [45] defined potential confounding factors (a priori) and made suitable adjustments for the treatment group and time since injury in their model. Although the treatments described in studies included in this review reflect current practice, most of these were not standardised and the nature of rehabilitation programmes such as neuromuscular training has been found to be correlated with better outcome [51]. Only one study [45] accounted for this confounding variable in their model. Two studies [47, 49] with a cohort from a randomised trial considered the mean effect of treatments administered, but did include it in their model because there was no difference between the groups.

Although we performed a comprehensive search strategy to reduce bias in our results, we did not perform hand searching of journals; hence, some studies that, generally, tend to be of poorer methodological quality may have been missed. We evaluated our studies using a robust quality assessment tool – QUIPS that covered all the important criteria for addressing the objectives of prognostic studies, which was pilot-tested to ensure consistency. However, a possible limitation in our approach at this stage was not performing an assessment of the inter-rater reliability for evaluating the quality of the studies.

Most factors identified exhibit a good degree of accessibility in clinical practice (See Table 5). The vast majority of the studies included in this review were of a short-term duration when symptoms are still severe and rapidly resolving, hence recovery at this stage is still quite variable. We identified a shortage of adequate prognostic studies evaluating predictors of recovery after acute ankle sprain at medium- (2-4 months) and long-term (≥4 months). Larger studies with adequate sample size per prognostic factor are also needed.

Furthermore, psychosocial and contextual factors such as recovery expectations, coping mechanisms, self efficacy, which have been implicated in recovery from musculoskeletal disorders [56] should be considered in future studies. We suggest that future studies consider the replication and confirmation of existing prognostic factors; exploring measures of internal and external validity; and adhere to current recommendations for conducting and reporting prognostic studies [57]. This will enable the translation of definitive prognostic factors into clinical practice. Overall, the existing evidence from the studies identified by this review does not allow firm conclusions to be drawn about prognostic factors of recovery from an acute ankle sprain.

## Conclusions

At present, the associations between baseline prognostic factors and recovery are largely inconsistent. Age seems to be an independent prognostic factor identified in three studies with consistent evidence for predicting recovery in patients with acute ankle sprain. However, we suggest a cautious interpretation due to the small associations between predictors and recovery. There is still some lack of clarity on the underlying mechanisms of recovery after an ankle sprain. More research is needed to inform an accurate understanding of the prognosis of acute ankle sprains.

### Clinical implications

- Factors that may be associated with poor recovery – at short-term include: pain intensity, difficulties bearing weight, restricted joint motion and functional ability.
- At long-term: older age, female gender.
- There is limited evidence that re-current sprain within 3 months, predicts subjective recovery at long term.
- Factors that were not investigated to date – psychosocial factors.
- There is a substantial gap in the literature for prognostic factors of poor recovery.

## Additional files

Additional file 1: Appendix A. Search strategy. (DOCX 26 kb)

Additional file 2: Appendix B. Criteria used for evaluating the quality of studies included in the systematic review using the QUIPS tool. (DOCX 17 kb)

## Abbreviations

AFS: Ankle Function Score
AMED: Allied and complementary medicine database
CINAHL: Cumulative index of nursing and allied health
FAOS: Foot and ankle outcome score
MRI: Magnetic resonance imaging
PRISMA: Preferred reporting items for systematic reviews and meta-analyses
QUIPS: Quality in prognosis studies
RCT: Randomised controlled trial
ROM: Restricted range of motion
SF36PF: 36-item short form survey physical functioning sub-domian
UK: United Kingdom
USA: United States of America
