# Supplementary material for: Prognostic factors for recovery following acute lateral ankle ligament sprain: a systematic review
Source: BMC Musculoskelet Disord. 2017 Oct 23;18:421. doi: 10.1186/s12891-017-1777-9 (PMC5654104; doi:10.1186/s12891-017-1777-9)
Supplement: Supplementary file 1 — Appendix A. Search strategy. (DOCX 26 kb) [file 12891_2017_1777_MOESM1_ESM.docx]

# Appendix A

**AMED (Allied and Complementary Medicine) via OVID**1985 to September 2016

1. exp Ankle/

2. ankle.ti,ab.

3. Calcaneus/

4. calcane$.ti,ab.

5. Talus/

6. talus.ti,ab.

7. talocrural.ti,ab.

8. talofibular.ti,ab.

9. calcaneofibular.ti,ab.

10. Ankle Joint/

11. (ankle adj joint$).ti,ab.

12. Tarsal Joint/

13. (tarsal adj joint$).ti,ab.

14. Tarsal bones/

15. (tarsal adj bone$).ti,ab.

16. (lateral adj1 ligament$).ti,ab.

17. OR/1-16

18. Ankle Injury/

19. (ankle adj injur$).ti,ab.

20. Sprains and Strains/

21. (sprain$ or strain$).ti,ab.

22. inversion.ti,ab.

23. OR/18-22

24. exp Prognosis/

25. prognos$.ti,ab.

26. predict$.tw.

27. exp Follow Up Studies/

28. (follow adj up adj stud$).ti,ab.

29. incidence.ti,ab.

30. course.ti,ab.

31. exp Longitudinal Studies/

32. longitudinal.ti,ab.

33. Prospective Studies/

34. prospect$.ti,ab.

35. Risk factors/

36. (risk adj factor$).ti,ab.

37. Cohort Studies/

38. (cohort adj stud$).ti,ab.

39. OR/24-38

40. 17 AND 23 AND 39

**CENTRAL Updated Search Strategy**

#1 Ankle :MH 1364

#2 ankle :TI,AB,KY 4530

#3 (Ankle Joint):MH 505

#4 (ankle joint*):TI,AB,KY 814

#5 (Tarsal Bones):MH 16

#6 (tarsal bones):TI,AB,KY 19

#7 (tarsal joint*):TI,AB,KY 12

#8 (Tarsal Joints):MH 10

#9 Calcaneus:MH 115

#10 calcane*:TI,AB,KY 353

#11 Talus:MH 20

#12 talocrural:TI,AB,KY 25

#13 talofibular:TI,AB,KY 9

#14 calcaneofibular:TI,AB,KY 10

#15 (Lateral Ligament, Ankle):MH 0

#16 (lateral ligament*):TI,AB,KY 96

#17 #1 OR #2 OR #3 OR #4 OR #5 OR #6 OR #7 OR #8 OR #9 OR #10 OR #11 OR #12 OR #13 OR #14 OR #15 OR #16 4835

#18 (Ankle Injury):MH 0

#19 (ankle injur*):TI,AB,KY 561

#20 (Ankle Sprain):MH 0

#21 (ankle sprain):TI,AB,KY 245

#22 (Sprains and Strains):MH 267

#23 (sprain* or strain*):TI,AB,KY 7127

#24 inversion:TI,AB,KY 582

#25 #18 OR #19 OR #20 OR #21 OR #22 OR #23 OR #24 7923

#26 Prognosis:MH 10961

#27 prognos*:TI,AB,KY 23331

#28 Forecasting:MH 463

#29 predict*:TI,AB,KY 51680

#30 (Follow Up):MH 48086

#31 follow?up*:TI,AB,KY 2075

#32 Incidence:MH 7849

#33 incidence:TI,AB,KY 59777

#34 (Cohort Studies):MH 6214

#35 (cohort stud*):TI,AB,KY 9473

#36 (Prospective Studies):MH 73954

#37 (prospect* stud*):TI,AB,KY 97763

#38 (Retrospective Studies):MH 6414

#39 (retrospect* stud*):TI,AB,KY 8809

#40 (Longitudinal Studies):MH 4966

#41 (longitudinal stud*):TI,AB,KY 6982

#42 (Risk Factors):MH 19329

#43 (risk factor*):TI,AB,KY 35375

#44 (Decision Support Techniques):MH 469

#45 #26 OR #27 OR #28 OR #29 OR #30 OR #31 OR #32 OR #33 OR #34 OR #35 OR #36 OR #37 OR #38 OR #39 OR #40 OR #41 OR #42 OR #43 OR #44 251623

#46 #17 AND #25 AND #45 324

#47 fracture:TI,AB,KY 7565

#48 #17 AND #25 AND #45 NOT 47 302

#49 01/01/2015 TO 27/07/2016:CD 118692

#50 #48 AND #49 33

**CINAHL via EBSCOHost – 1982 to September 2016**

1. MH Ankle
2. TI ankle* OR AB ankle*
3. TI calcaneofibular OR AB calcaneofibular
4. TI talofibular OR AB talofibular
5. TI talocrural OR AB talocrural
6. TI (ankle N1 joint*) OR AB (ankle N1 joint*)
7. TI “tarsal joint*” OR AB “tarsal joint*”
8. TI “tarsal bone*” OR AB “tarsal bone*”
9. MH Calcaneus
10. MH Talus
11. MH Tarsal Bones+
12. MH Lateral Ligament, Ankle
13. TI (lateral N1 ligament) OR AB (lateral N1 ligament)
14. MH Ankle Sprain
15. MH Sprains and Strains
16. TI sprain* OR AB sprain*
17. TI strain* OR AB strain*
18. MH Ankle Injuries
19. TI (injur* N1 ankle) OR AB (injur* N1 ankle)
20. TI (inversion N1 sprain*) OR AB (inversion N1 sprain*)
21. MH Incidence
22. TI predict* OR AB predict*
23. TI “cohort stud*” OR AB “cohort stud*”
24. TI course OR AB course
25. MH Predictive research
26. MH Prognosis
27. TI prognos* OR AB prognos*
28. TI “follow up stud*” OR AB “follow up stud*”
29. TI “follow-up stud*” OR AB “follow-up stud*”
30. MH Prospective studies+
31. TI “longitudinal stud*” OR AB “longitudinal stud*”
32. MH Risk Factors
33. TI recovery OR AB recovery
34. TI (treatment N1 outcome*) OR AB (treatment N1 outcome*)
35. S1 OR S2 OR S3 OR S4 OR S5 OR S6 OR S7 OR S8 OR S9 OR S10 OR S11 OR S12 OR S13
36. S14 OR S15 OR S16 OR S17 OR S18 OR S19 OR S20
37. S21 OR S22 OR S23 OR S24 OR S25 OR S26 OR S27 OR S28 OR S29 OR S30 OR S31 OR S32 OR S33 OR S34
38. S35 AND S36 AND S37 retrieved 194 articles / 204 articles on the 26^th^ july 2016

**EMBASE via Ovid - 1974 to September 2016**

1. exp Ankle/
2. ankle.ti,ab.
3. Ankle Lateral Ligament/
4. (ankle adj lateral adj ligament).ti,ab.
5. Calcaneus/
6. calcane$.ti,ab.
7. Talus/
8. talus.ti,ab.
9. calcaneofibular.ti,ab.
10. talofibular.ti,ab.
11. talocrural.ti,ab.
12. (ankle adj joint$).ti,ab.
13. Tarsal Joint/
14. (tarsal adj joint$).ti,ab.
15. OR/1-14
16. Ankle Sprain/
17. Sprain/
18. sprain$.ti,ab.
19. strain$.ti,ab.
20. (inversion adj sprain$).ti,ab.
21. Ankle Injury/
22. OR/16-21
23. follow-up.mp.
24. prognos:.tw.
25. ep.fs.
26. OR/23-25
27. 15 AND 22 AND 26

**OpenGREY search strategy**

Simple search in titles and abstracts for “ankle sprain or ankle”

**PEDro search strategy**

Simple search in titles and abstracts for “ankle sprains”

**PsycINFO via Ovid – 1806 to September 2016**

1. exp Ankle/

2. ankle.ti,ab.

3. (ankle adj lateral adj ligament).ti,ab.

4. calcane$.ti,ab.

5. talus.ti,ab.

6. calcaneofibular.ti,ab.

7. talofibular.ti,ab.

8. talocrural.ti,ab.

9. (ankle adj joint$).ti,ab.

10. (tarsal adj joint$).ti,ab.

11. OR/1-10

12. sprain$.ti,ab.

13. strain$.ti,ab.

14. inversion.ti,ab.

15. OR/12-14

16. Prognosis/

17. prognos$.ti,ab.

18. predict$.ti,ab.

19. Followup Studies/

20. (follow?up adj stud$).ti,ab.

21. incidence.ti,ab.

22. course.ti,ab.

23. Longitudinal Studies/

24. (longitudinal adj stud$).ti,ab.

25. Prospective Studies/

26. (prospective adj stud$).ti,ab.

27. Risk Factors/

28. (risk adj factor$).ti,ab.

29. Cohort Analysis/

30. (cohort adj stud$).ti,ab.

31. Disease course/

32. OR/16-32

**PubMed search strategy – 26^th^ September 2016**

Ankle [mh]
2. ankle* [tiab]
3. Lateral Ligament, Ankle [mh]
4. calcane* [tiab]
5. Ankle Joint [mh]
6. ankle joint* [tiab]
7. tarsal joint* [tiab]
8. calcaneofibular [tiab]
9. talofibular [tiab]
10. talocrural [tiab]
11. talus [tiab]
12. #1 OR #2 OR #3 OR #4 OR #5 OR #6 OR #7 OR #8 OR #9 OR #10 OR #11
13. Ankle Injuries [mh]
14. sprain* [tiab]
15. strain* [tiab]
16. Sprains and Strains [mh]
17. inversion [tiab]
18. #14 OR #15 OR #16 OR #17

19. Prognosis [MeSH:noexp]
20. diagnosed [tiab]
21. cohort* [tiab]
22. Cohort effect [mh]
23. Cohort studies [MeSH:noexp]

24. predictor* [tiab]
25. death [tiab]
26. "models, statistical" [mh]

27. #19 OR #20 OR #21 OR #22 OR #23 OR #24 OR #25 OR #26

28. #12 AND #18 AND #27

**SportDiscus via EBSCOHost 1966 -2016**

1. SU Ankle

2. TI ankle* OR AB ankle*

3. TI calcaneofibular OR AB calcaneofibular

4. TI talofibular OR AB talofibular

5. TI talocrural OR AB talocrural

6. TI “ankle joint*” OR AB “ankle joint*”

7. TI “tarsal joint*” OR AB “tarsal joint*”

8. TI “tarsal bones” OR AB “tarsal bones”

9. TI calcane* OR AB calcane*

10. TI talus OR AB talus

11. SU Ankle Lateral Ligament

12. TI “lateral ligament” OR AB “lateral ligament”

13. S1 OR S2 OR S3 OR S4 OR S5 OR S6 OR S7 OR S8 OR S9 OR S10 OR S11 OR S12

14. SU Sprains

15. SU Strain

16. TI sprain* OR AB sprain*

17. TI strain* OR AB strain*

18. TI (injur* N1 ankle) OR AB (injur* N1 ankle)

19. TI (inversion N1 sprain*) OR AB (inversion N1 sprain*)

20. S12 OR S13 OR S14 OR S15 OR S16 OR S17 OR S18 OR S19

21. TI incidence OR AB incidence

22. TI predict* OR AB predict*

23. TI course OR AB course

24. TI cohort* OR AB cohort*

25. TI “cohort stud*” OR AB “cohort stud*”

26. SU Prognosis

27. TI prognos* OR AB prognos*

28. TI “follow up stud*” OR AB “follow up stud*”

29. TI “follow-up stud*” OR AB “follow-up stud*”

30. TI “longitudinal stud*” OR AB “longitudinal stud*”

31. TI “risk factor*” OR AB “risk factor*”

32. TI forecasting OR AB forecasting

33. TI “decision making” OR AB “decision making”

34. TI predict* and AB predict*

35. SU Cohort analysis

36. S21 OR S22 OR S23 OR S24 OR S25 OR S26 OR S27 OR S28 OR S29 OR S30 OR S31 OR S32 OR S33 OR S34 OR S35

36. S11 AND S20 AND S36
