# Supplementary material for: Prognostic factors for recovery following acute lateral ankle ligament sprain: a systematic review
Source: BMC Musculoskelet Disord. 2017 Oct 23;18:421. doi: 10.1186/s12891-017-1777-9 (PMC5654104; doi:10.1186/s12891-017-1777-9)
Supplement: Supplementary file 2 — Appendix B. Criteria used for evaluating the quality of studies included in the systematic review using the QUIPS tool. (DOCX 17 kb) [file 12891_2017_1777_MOESM2_ESM.docx]

**Table 2 – Criteria used for evaluating the quality of studies included in the systematic review using the QUIPS tool**

**Section one: sampling of patients / external validity**

1. Inclusion criteria are clearly defined
2. Exclusion criteria are well defined
3. Source population was well defined
4. Adequate description of diagnostic criteria for classifying patients with ankle injury
5. Clinical and demographic characteristics are fully described and reproducible
6. The sample is representative of the majority of patients with an acute ankle sprain
7. The sample is assembled at a common point (within a one week of sustaining the injury)
8. The sample is complete
9. Any treatment received is fully described (including no treatment).

**Section two: measurements used / internal validity**

1. Outcomes measured with established test-retest reliability in a similar population have been used.
2. Study assessors were blinded to baseline and outcome data.
3. Prognostic factors are fully defined, including details of method used for conducting the measurements
4. Measurements used for the prognostic factors are standardised or validated

**Section three: statistical analysis**

1. Was the sample size adequate for the number of prognostic factors included in the analysis (minimum of 10 per factor)?
2. Loss to follow up is < 20% or methods used to handle missing data.
3. Was multivariate analysis carried out?
4. Statistical adjustment for selecting important prognostic factors, including age, re-sprain, pain and instability; which are clearly defined a prior.

**Section four: evaluation of the model**

1. Internal (using the data from which the model was developed) and external (prediction developed from patients who were not used to produce the model) validation of the final model.
2. Clinical utility and effect of the model in clinical practice
3. The ease of use of the model – accessibility of the data used to generate the model, clear descriptions of the actual model and coding procedures used and corresponding confidence intervals.
